# Metabolic Brain Disorders: Prodromes, Symptoms, and Syndromes

**DOI:** 10.3390/ijms27125190

**Published:** 2026-06-08

**Authors:** Janusz Wiesław Błaszczyk

**Affiliations:** Department of Human Behavior, Jerzy Kukuczka Academy of Physical Education, 40-065 Katowice, Poland; januszwblaszczyk@gmail.com

**Keywords:** brain, energy metabolism, aging, neurodegenerative disorders, information metabolism

## Abstract

Life is a self-organizing and self-sustaining process that involves energy transformation, primarily regulated by the brain. The brain’s main structure consists of terminally differentiated, postmitotic, non-replaceable cells, whose proper functioning and longevity depend solely on glucose-based energy metabolism. Glucose serves as the primary substrate for cellular respiration and anaerobic processes, which are essential for maintaining proper neuronal function, homeostasis, and cell repair. Research indicates that brain aging and neurodegenerative changes result from an age-related decline in glucose metabolism, largely due to a deficiency in nicotinamide adenine dinucleotide (NAD). This deficiency is particularly harmful to brain structures that contain neurons with the highest energy demands. The first signs of brain aging typically appear in the hypothalamus, as well as in the GABAergic and glutamatergic structures of the cerebral cortex and subcortical nuclei. Early symptoms of senile brain changes often manifest as systemic metabolic disorders like insulin resistance and type 2 diabetes. These are accompanied by alterations in brain energy metabolism, leading to neurological and psychiatric disorders that correspond to the affected brain regions. Over time, these changes gradually impact the brain’s regions with the highest energy consumption. Current clinical studies suggest that early supplementation with NAD precursors may help slow the aging and neurodegeneration processes. However, this protective therapy appears to be less effective once the disease is fully developed.

## 1. Introduction

Life is a self-organizing and self-sustaining process that involves the transformation of energy, primarily regulated by the brain. A fundamental condition for these energy processes is the organism’s adaptation to its environment. To achieve this, a living organism learns about its surroundings through its senses. The sensory patterns it acquires, along with the corresponding responses to the environment, are stored in the brain’s memory structures, which shape our behavior.

**Figure 1:**
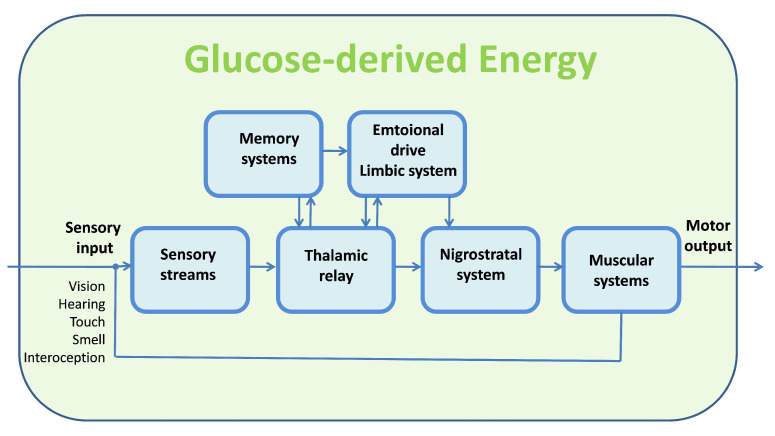
A simplified flowchart illustrates information and energy metabolism in the human brain. Sensory signals are transmitted to the thalamus, which, under the guidance of memory and emotional structures, uses these signals to activate muscular executive systems that implement essential life behaviors. The overall activity and efficiency of the entire neuromuscular system rely on both aerobic and anaerobic glucose metabolism.

The processes involved and the behaviors they generate are referred to as “information metabolism” [1]. This term encompasses the mental processes of organizing, identifying, and interpreting sensory information to understand the environment. To facilitate this, the brain uses various neurotransmitters and neuromodulators to assign emotional and motivational values—positive or negative—to the sensory information it perceives. Information metabolism enables the exchange of information between a living organism and its environment, aiming to preserve its identity and structure. Self-awareness is considered the highest form of information metabolism and is associated with several important behavioral markers, such as arousal, attention, facial expressions, body movements, and task engagement (Figure 1).

The thalamic reticular nucleus (TRN) serves as a key modulator and controller of information flow between the thalamus and cortex, playing significant roles in attention, alertness, sleep regulation, and sensory filtering [2]. The brain regulates the flow of sensory information from the environment to the cortex by modulating the inhibition of parvalbumin-positive GABAergic neurons in the TRN [2]. The parvalbumin neuronal network of the TRN also mediates the recognition of familiar individuals. Within this network, GABA release is regulated by the posterior parietal cortex-sensory reticular thalamus-parafascicular thalamic nucleus circuit, which is involved in social memory [3]. Social memory, the ability to remember close individuals, is crucial for maintaining stable, positive social relationships, including social hierarchy, defensive behaviors, cooperation, and mating [3,4]. Social memory intertwines with various streams of sensory cues, including visual, olfactory, and auditory signals, enabling individuals to adapt their behavior to the social environment [3,4].

Recent evidence links thalamic inhibition and its disruption to attentional deficits and disturbed sleep patterns observed in schizophrenia [5]. The thalamus integrates sensory and motor information, which helps maintain alertness and wakefulness [6,7]. It is the primary subcortical structure supplying signals to the neocortex [8]. The thalamus transmits almost all sensory information (except for olfaction) to the cerebral cortex, regulating consciousness, sleep, attention, and motor functions. The basal dendrites of neocortical pyramidal neurons receive most of these signals [8]. Additionally, the thalamus receives projections from the limbic system, which supports the processing and management of emotions as well as the formation and storage of emotionally labeled memory traces. Higher-order thalamocortical projections convey information about the overall state of arousal, providing contextual cues that can flexibly modulate cortical sensory processing [6,7].

The brainstem ascending arousal system is responsible for regulating wakefulness, alertness, and consciousness through various neurotransmitters, including norepinephrine, serotonin, acetylcholine, and dopamine [9]. During sleep, the thalamus ceases to transmit external sensory input, and its neurons begin to pulse rhythmically, initiating synchronized cortical oscillations. These oscillations play a crucial role in the dynamic integration of cortical memory networks [9]. Dysfunctions in specific neurotransmitter systems can disrupt thalamocortical control, resulting in a wide range of symptoms. Such dysfunctions are associated with sensory abnormalities, attention deficits, and sleep disturbances across multiple disorders. A failure in cortico-thalamo-cortical transmission has been linked to the neurobiology of schizophrenia [10]. This mental illness significantly impacts an individual’s cognition, emotions, and social interactions, often characterized by hallucinations, delusions, thought disorders, attentional deficits, apathy, and frequently disturbed sleep [5].

An essential aspect of consciousness is the perception of one’s environment, which is recorded in the brain as an egocentric representation. These perceived egocentric representations are stored in primary sensory cortex areas [3], while allocentric representations, which are defined concerning the external world, are stored in the hippocampus, entorhinal cortex, and adjacent areas [3,11]. The formation of these representations is a continuous process, relying on streams of sensory information from exteroceptors (vision, hearing, smell) and interoceptors that are activated during motor activities and transmitted to the cortex via the thalamus. The perceived egocentric representations are again recorded in the primary sensory cortex areas [3], while allocentric representations are cataloged in the hippocampus and related regions [3,11]. Both spatial memory and spatial orientation experience a gradual yet noticeable decline due to neurobiological changes and a general slowdown in cognitive functions [12]. Both motor and cognitive functions are particularly vulnerable to deterioration with age, a symptom commonly associated with Alzheimer’s disease [12].

## 2. Energy Metabolism, Mood, Emotions, and Motivation

Energy deficiency poses a significant threat to life, acting as a major stressor for the body. In response to this deficit, the brain may exhibit symptoms such as low mood, increased anxiety, and aggressive behaviors. Meeting hunger needs and restoring energy levels activate the brain’s reward system, ultimately enhancing overall well-being. Emotional balance relies on the coordinated activity of several brain regions, particularly the prefrontal cortex and the amygdala. When these systems are disrupted by stress, illness, or metabolic issues, emotional states can become unstable or negative. Notably, energy metabolism and emotional regulation are closely linked, and disruptions in either process can lead to decreased motivation. Low or inefficient energy use can hinder mood regulation, concentration, and motivation. Motivation is significantly impaired in individuals with depression, reflecting actual changes in the brain systems responsible for drive and reward. The primary cause of a low mood may be an energy deficit and related changes in the motivational-emotional system. A key symptom of low mood is anhedonia, or the inability to experience pleasure.

The key brain structure involved in mitigating fear and anxiety is the dopaminergic system of the nucleus accumbens, which plays a crucial role in the experience of reward, pleasure, and motivation. The nucleus accumbens functions as a limbic-motor interface, transforming emotional stimuli from the limbic system into behaviors executed by the motor system. Various types of social interactions engage the nucleus accumbens, and insufficient or poor social relationships are often linked to several mental disorders, including depression, schizophrenia, and anxiety disorders [13]. To alleviate stress, oxytocinergic neurons located in the paraventricular, supraorbital, and accessory nuclei of the hypothalamus produce the neuropeptide oxytocin [14].

Fear is an acquired response to aversive stimuli, while anxiety is a chronic state characterized by a low mood, indicating the body’s struggle to cope with real or imagined threats. Both fear and anxiety are mediated by different regions in the brain [15]. Fear responses are linked to activity in the central nucleus of the amygdala, whereas state anxiety is governed by the central nucleus of the bed nucleus of the stria terminalis (BNST). The BNST acts as a relay center in neural circuits, coordinating the functions of the autonomic, neuroendocrine, and somatic motor systems to achieve organized physiological responses and behaviors [15].

Oxytocin receptor activity in the BNST is important for fear reduction through social recognition [16]. The release of oxytocin is triggered by numerous stimuli, such as positive interactions with humans or animals, suckling, food intake, light skin stimulation, and sexual and maternal behaviors. When pleasant sensory stimulus occurs, oxytocin contributes to anti-stress effects, which include lowered blood pressure and cortisol levels [14,17]. Under these circumstances, oxytocin can reduce stress, anxiety, and depression, inhibit compulsive behaviors, and generally enhance social interactions. Additionally, oxytocin exhibits anti-inflammatory effects in the brain, suggesting potential therapeutic applications in aging and neurodegenerative diseases [14].

BNST serves as a hub where descending information from the cortex meets ascending inputs about homeostatic states or potential changes in homeostasis [18]. The BNST integrates stress, fear, anxiety, and reward stimuli from the amygdala, hypothalamus, and brainstem [19]. This sexually dimorphic structure comprises 12 to 18 subnuclei, each containing distinct neuronal populations that project to areas such as the septal area, hypothalamus, cerebral cortex, and thalamus [19].The anterior subpopulation seems to focus on energy balance, while the posterior group may be more involved in reproductive and defensive behaviors [18,19]. The BNST is also central to the psychogenic brain circuit that connects the hippocampus with the paraventricular nucleus, a primary regulator of the hypothalamic–pituitary–adrenal axis [19]. In individuals with anxiety disorders, altered threat responses are observed, marked by heightened amygdala activity at the onset of threat anticipation and sustained activation of the BNST thereafter.

Emotion significantly influences cognitive processes in humans, including perception, attention, learning, memory, reasoning, and problem-solving [20]. The dorsal striatum is crucial for making decisions, particularly about action selection and initiation, through the integration of sensorimotor, cognitive, and motivational/emotional cues within specific glucocorticoid (cortisol) signaling pathways. While the ventral striatum and ventral tegmental area (VTA) are responsible for reward-related learning, the medial temporal lobe is involved in fear-related learning [21]. The central extended amygdala serves as a key circuit for emotional processing and stress responses, with its components containing GABAergic neurons activated by various neurotransmitters [22]. Dysfunction within the amygdalar complex is strongly linked to anxiety disorders and depression [22].

A depressed mood alters the activity of the prefrontal cortex, diminishing its ability to initiate and sustain actions that may seem impossible under conditions of energy deficit. Furthermore, excessive activity in areas like the amygdala can promote negative emotions and depression, often creating a vicious cycle: lower motivation leads to reduced activity, fewer satisfying experiences, and deeper depression. Chronically low energy balance forces the brain to switch from utilizing external energy sources to relying on internal ones, primarily through gluconeogenesis, which restores blood sugar levels to maintain essential brain function. This shift in nutrient utilization from primarily glucose to other sources is a fundamental metabolic adaptation necessary to cope with decreased blood glucose levels and a decline in glucose oxidation. AMP-activated protein kinase (AMPK) plays a crucial role in this metabolic adaptation [23].

The lateral septum is a brain structure that regulates food intake by integrating homeostatic and motivational stimuli [24]. Anatomically, the lateral septum connects the hippocampus, which is essential for memory and spatial orientation, with various subcortical areas, particularly those in the hypothalamus that play a crucial role in motivated behaviors necessary for survival, such as eating, arousal, energy homeostasis, and reward-related actions. The septum’s projection to the lateral preoptic area is involved in regulating sleep–wake cycles, thermoregulation, and reward-seeking motor behaviors, including locomotion and food consumption. The lateral septum, which is composed almost entirely of GABAergic neurons, is activated by glucagon-like peptide-1, a gut hormone essential for regulating blood sugar levels and feelings of fullness. These neurons express somatostatin, leading to the inhibition of growth hormone, insulin, glucagon, and calcium-dependent protein secretion. The activity of the lateral septum is modulated by several neurotransmitters and neurohormones, including vasopressin, oxytocin, ghrelin, corticotropin-releasing factor (CRF), and particularly neuropeptide Y.

## 3. Brain Memory System Is the Major Consumer of Energy

Information processing in the cerebral cortex and hippocampus is influenced by network oscillations that vary in frequency depending on brain state and behavior. In the hippocampus, theta oscillations (4–10 Hz) are present during exploratory behavior and rapid eye movement (REM) sleep. Meanwhile, short bursts of high-frequency activity (120–200 Hz), which last about 100 ms, are typical during slow-wave sleep, waking inactivity, and consummatory behavior [25]. The physiological functions of the brain are determined by the integration of biochemical signals from primary neurotransmitters, specifically glutamate and gamma-aminobutyric acid (GABA).

**Figure 2:**
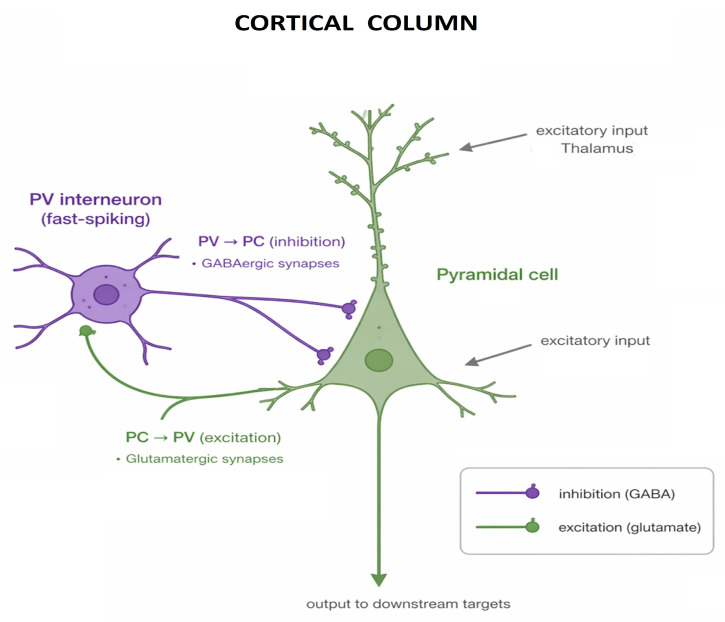
Basic structure of the cortical glutamatergic and GABAergic systems. The balance of glutamate and gamma-aminobutyric acid (GABA) signaling is crucial for cognition, memory, and neuroplasticity. Pyramidal neurons (PC) receive excitatory sensory signals from the thalamus, while the parvalbumin-positive interneurons (PV) form a dense inhibitory network that strongly controls pyramidal neuron activity by generating high-frequency spikes. This allows for synchronization of the cortical column network and the generation of gamma oscillations (30–80 Hz).

Parvalbumin, a calcium-binding protein found in rapidly responding GABAergic neurons, protects these neurons from calcium-induced apoptosis by regulating calcium ion levels. Additionally, various neuromodulators, neurohormones, and ionic signaling further modulate the activity and function of the glutamatergic and GABAergic systems (Figure 2). Glutamate-mediated synaptic transmission accounts for 80–90% of total glucose use in the cerebral cortex, particularly in areas with the highest synaptic activity [26]. In the human cerebral cortex, parvalbumin-positive neurons form a dense inhibitory network that strongly influences the activity of pyramidal neurons [27]. Thanks to parvalbumin, the GABA interneurons in pyramidal columns can generate very rapid pulse trains in response to calcium signaling. Their inhibitory function allows for the synchronization of neural networks and the generation of gamma oscillations (30–80 Hz), which are critical for cognitive functions, memory, and neuroplasticity [28]. This high level of activity and metabolism results in significant energy demands [28]. Therefore, GABA interneurons are particularly sensitive to stress and pathological factors.

Damage or dysfunction of these interneurons is strongly associated with neuropsychiatric disorders, including schizophrenia and autism [27,29]. Each pyramidal neuron receives inhibitory inputs from multiple nearby interneurons, providing redundancy that enhances the functionality and reliability of cortical memory networks (Figure 1). However, the intense activity of parvalbumin (PV) interneurons requires a high level of energy metabolism, making them more susceptible to disruptions. Parvalbumin-expressing interneurons are especially vulnerable to stressors and have been linked to various neuropsychiatric diseases. Injury to or dysfunction of PV inhibitory interneurons is associated with the pathophysiology of several significant neuropsychiatric disorders, including schizophrenia, autism spectrum disorder, bipolar disorder, various neurodegenerative diseases, and even age-related cognitive decline [27].

As individuals age, the function of PV interneurons tends to decline, which can worsen cognitive and memory deficits. This decline is often due to impaired dopamine and glutamate signaling. Inflammation and chronic stress may also affect parvalbumin expression in the prefrontal cortex, potentially leading to behavioral and cognitive deficits. Elevated cortisol levels have been shown to reduce parvalbumin expression and contribute to cognitive and emotional impairments observed in conditions such as chronic stress, post-traumatic stress disorder, and depression [29]. One critical factor that increases the susceptibility of cortical neurons to damage is the energy-intensive process of neural network formation, which relies on tight integration among neurons, astrocytes, and glial cells [30].

Synaptic pruning is a physiological mechanism essential for the proper formation and maturation of functional neural networks; it eliminates rarely used synapses while preserving frequently used connections [31]. Although pruning occurs throughout life, it is particularly extensive in brain regions associated with higher cognitive functions during adolescence. Importantly, in a healthy brain, apoptotic synapses undergo local biochemical changes typical of apoptosis without leading to the death of the entire neuron. This allows microglia and astrocytes to selectively identify and eliminate weakened or redundant synapses [31]. In the case of overactive synapses and neurons, excessive calcium ion influx triggers the externalization of phosphatidylserine, which activates phagocytosis.

In addition to synaptogenesis and synaptic pruning, neurogenesis in the adult brain is another memory-forming process. However, its level and changes across the lifespan are not yet fully understood. Neurons in the cerebral cortex do not undergo replacement through cell turnover. When these neurons die, there is a slow but irreversible decline in cognitive and executive functions. Only a small population of interneurons is regularly replaced in several brain structures, including the hippocampus, striatum, olfactory bulb, and potentially the cerebellum. These structures are associated with memory, motor behavior, and emotional control; their neurodegeneration leads to specific behavioral changes and the development of neural and emotional disorders. Significant amounts of energy are required for neural processes related to learning and memory, particularly for synaptogenesis and neurogenesis. In humans, adult neurogenesis occurs only temporarily in specific brain regions associated with learning and memory, including the hippocampus, olfactory bulb, striatum, and possibly the cerebellum [32,33,34]. The fate of stem cells—regarding their division, differentiation, migration, and integration into existing neural networks—depends on glutamine-dependent ATP production. Thus, glutamine is essential for effective neurogenesis [35,36].

In the mature human brain, neurogenesis probably involves only GABA interneurons and glial cells, with the highest intensity of these processes observed in the early stages of development [37]. Neurogenesis-based memory formation allows individuals to adapt their personality and behavior to environmental demands [36]. Each new experience is integrated into existing memory networks as patterns of sensory and motor components, associated with contextually specific positive or negative emotional signatures. During adult neurogenesis, activated progenitor cells must be incorporated into existing memory networks, facilitating their terminal differentiation and establishing presynaptic and postsynaptic interactions with existing neurons, glial cells, and the extracellular matrix.

Circulation of the cerebrospinal fluid facilitates progenitor cell migration during adult neurogenesis [33,38]. These processes depend on the quality and stability of cell membranes and the physiology of the intercellular space [39]. Particularly, the outer layer of glycans on cell membrane surfaces, known as the glycocalyx, plays a significant role in sensing and transducing biophysical signals—specifically, mechanical forces acting on cells that elicit cellular responses [40,41]. Sialic acid, a monosaccharide with high polarity and a negative electrical charge, forms a thin electrostatic layer on the surfaces of cell membranes, mitochondrial membranes, endoplasmic reticulum, and microtubules. In the brain, sialic acid is found in glycoproteins, glycolipids, and interstitial fluids, making it crucial for morphogenesis, cell recognition and adhesion, immunity, and neurotransmission. Sialic acid-containing glycosphingolipids are potent bioactive signaling molecules that regulate cell growth, differentiation, apoptosis, and inflammation [42]. Siglecs are key microglial receptors that recognize sialic acid on healthy neurons, helping to prevent excessive inflammation and maintain tissue homeostasis while also responding to waste products.

Neu5Ac sialic acid plays a crucial role in the plasticity of the adult brain, serving as a key structural modulator that facilitates the remodeling of neuronal connections and intercellular spaces [43]. Due to its physicochemical properties and high negative electrical charge, polysialic acid generates repulsive forces between cell membranes. This reduces adhesion between neurons, creating a flexible environment that is essential for the formation of new synapses while allowing for the removal of old ones [43]. Extensive polysialylation is found in brain structures known for their high plasticity, such as the hippocampus and olfactory bulbs. Neu5Ac sialic acid is the sole ligand for microglial Siglecs, and interactions between sialic acid and Siglecs are believed to play a critical role in regulating microglial homeostasis in a healthy brain [44]. Deficient polysialylation is a significant factor behind impaired migration of nervous system stem cells, which negatively impacts neurogenesis. As individuals age, levels of polysialic acid in the brain decline, leading to reduced plasticity and regenerative capacity [43].

Brain-derived neurotrophic factor (BDNF) plays a vital role in the development of brain circuits, the formation and maintenance of neuronal morphology and architecture, and synaptic plasticity, ultimately regulating learning and memory processes [45]. BDNF is primarily released from neurons in an activity-dependent manner, particularly through NMDA receptors and increased calcium influx, which promotes vesicle fusion and release [45]. This release allows BDNF to act on local TrkB receptors, strengthening neural connections [45]. Additionally, BDNF is released from vascular endothelial cells, which contribute to the vascularization of neuronal structures. BDNF levels in preterm neonates differ from those in full-term neonates, influencing cognitive development in early postnatal life and potentially being linked to mental health issues in children, such as autism spectrum disorders. In the adult brain, imbalances in BDNF levels and downstream signaling via its cognate TrkB tyrosine kinase receptor are associated with neurodegenerative and psychiatric diseases, including Alzheimer’s disease, major depressive disorder, and schizophrenia [45]. Trophic factors—such as BDNF, fibroblast growth factor (FGF), epidermal growth factor (EGF), and the neurotransmitter serotonin—are involved in enhancing adult neurogenesis [46]. Both acute and chronic stress are known to decrease hippocampal neurogenesis. However, persistent hippocampal neurogenesis is observed in older adults, including those in their 10th decade of life, and in patients with mild cognitive impairments and Alzheimer’s disease.

The hippocampus plays a vital role in learning by maintaining a neurogenic reserve, particularly when individuals encounter new and complex experiences. Young adults typically exhibit a high level of neurogenesis, which is the process of generating new neurons, due to their active adaptation to their environment. However, neurogenesis declines significantly with age, peaking around puberty [46]. Under conditions of glutamine deficiency, stem cells rapidly die due to ATP depletion, resulting in impaired connectivity in the memory-related neuronal networks of older adults [47]. Adult neurogenesis is now recognized as a form of brain plasticity that contributes to various physiological functions and impairments in this process are linked to neurological and psychiatric disorders [48]. Anxiety often serves as a precursor to mental health issues and is associated with reduced neurogenesis and cognitive deficiencies in humans.

Newly formed neurons in the hippocampus express glucocorticoid receptors, and any dysregulation of the hippocampus-hypothalamic–pituitary–adrenal (HPA) axis can hinder memory and learning. Stress and depression have also been correlated with decreased neurogenesis. Some studies suggest that the decline in neurogenesis can be mitigated by the use of antidepressants, antipsychotics, and/or increased physical exercise [46]. Neurogenesis is supported by microglia, but in an aging brain, microglial activity is characterized by pro-inflammatory cytokines that inhibit neurogenesis, which in turn impairs learning and memory [49]. As a result, cognitive performance in older adults declines due to limited neurogenesis, and learning and memory processes are restricted to the restructuring of neural networks by changing the number and strength of synaptic connections [37]. The hippocampus is critical for cognitive processes, especially in the initial formation of memory traces, while the neocortex serves as the ultimate storage area for these memories. Consequently, the most energy-demanding cortical areas include the medial temporal lobe and frontal regions, particularly when maintaining sensory representations in working memory. Dysfunction in memory processes is central to conditions such as temporal lobe epilepsy and Alzheimer’s disease, with atrophy often extending beyond the hippocampus to involve surrounding structures [50].

## 4. Brain Energy Metabolism

The organism constantly absorbs energy from its surroundings to maintain its structure and homeostasis, facilitate self-repair, and engage in motor behaviors. The hypothalamus plays a crucial role in maintaining physiological, behavioral, and energy homeostasis [51]. It serves as the primary regulatory center for body temperature, hunger, thirst, sleep–wake cycles, circadian rhythms, behavior, and hormone secretion [52]. These regulations are closely linked to mood control, motivation, and stress response. Impaired glucose metabolism in the aging body can lead to dysregulation of the hypothalamic–pituitary–adrenal (HPA) axis, the hypothalamic–pituitary–thyroid (HPT) axis, and the hypothalamic–pituitary–gonadal (HPG) axis.

These disturbances often manifest in the early stages of neurodegenerative diseases [53]. Even mild but prolonged energy deficiency can manifest with nonspecific prodromal symptoms, such as increased anxiety and memory impairment. Dysfunction of the hypothalamic–pituitary–adrenal (HPA) axis, the hypothalamic–pituitary–thyroid (HPT) axis, and the hypothalamic–pituitary–gonadal (HPG) axis has been consistently observed in patients with Alzheimer’s disease and may contribute to the pathophysiological development of this condition [53]. Amyloid plaques and NFTs have been observed in the suprachiasmatic nucleus, paraventricular nucleus, lateral hypothalamic area, tuberomammillary nucleus, and optic nucleus [53].

All physiological processes that support homeostasis rely on providing neurons with the necessary substrates for energy metabolism and efficiently removing metabolic waste products. The movement of cerebrospinal fluid is crucial for brain functions, including the clearance of excess fluid and metabolic waste [54,55]. The glymphatic system aids in the local and global transport of signaling molecules and metabolites [55]. The brain is equipped with a complex circulatory system, consisting of vascular and glymphatic subsystems, which supplies glucose, oxygen, and nutrients while also removing metabolic waste products. This system is crucial for adult neurogenesis, the migration of progenitor cells, and the formation of neural networks related to memory processes. The structure and functional state of the blood–brain barrier are pivotal to brain function and the overall health of the organism. It acts as the primary defense, protecting the nervous system from toxins, pathogens, and unwanted metabolites that could impair brain function.

Astrocytes form a metabolic network connecting neurons and blood vessels, thereby supplying neurons with nutrients, removing metabolic waste, regulating blood flow to the central nervous system, and maintaining the integrity of the blood–brain barrier. This barrier helps stabilize the composition of cerebral blood circulation, making it less susceptible to systemic fluctuations. The mechanisms of neurovascular coupling are multifaceted, involving mediators released from different cells that activate distinct signaling pathways across the entire cerebrovascular network in a highly coordinated manner [56]. Various brain structures, such as the subfornical organ and vascular organ of the lamina terminalis, as well as the periventricular sensory organs in the forebrain and posterior hindbrain regions, play roles in regulating systemic homeostasis [57]. However, these structures lack a tight blood–brain barrier, which exposes their neurons to toxins, pathogens, and elevated concentrations of signaling ions, particularly calcium and phosphate from the blood. This vulnerability makes them more susceptible to aging, neurodegeneration, and cell death (apoptosis).

In the aged, reduced cerebral blood flow and abnormal cerebrovascular reactivity significantly impair brain function. This leads to the accumulation of waste products in neurons and the interstitial space, disrupting homeostasis and the kinetics of biochemical reactions. Calcium ions, distributed by blood flow, play a crucial role in regulating major physiological processes in the brain, controlling neurotransmitter release, synaptic plasticity, and growth cone motility. The blood–brain barrier is permeable to gases (such as oxygen and carbon dioxide), glucose, and ions essential for maintaining the resting and functional potentials of nerve cells. Thus, the blood–brain barrier is vital for sustaining homeostasis and physiological processes in the central nervous system. In neurons, calcium influx through NMDA receptors and voltage-dependent calcium channels mediates several physiological processes; however, impaired calcium signaling contributes to neuronal degeneration and death in neurodegenerative conditions [58].

The brain, which serves as the controller of life, requires a constant supply of glucose and oxygen from the blood [59]. Under normal physiological conditions, glucose and, to a limited extent, fatty acids are the primary energy sources [60,61]. Glucose metabolism in the brain is closely tied to all life processes, including the regulation of neuroenergetics, neurotransmission, biosynthesis, and antioxidant defense [59]. The primary pathways for energy production in neurons are glycolysis and oxidative metabolism, which occur via the tricarboxylic acid cycle and the electron transport chain. The entry of neuroactive compounds essential for protein synthesis and energy production, such as glutamate, aspartate, glycine, and D-serine, into the brain is strongly restricted by the blood–brain barrier; therefore, these compounds are synthesized from glucose within the brain [62]. During energy metabolism, these neuromodulators and neurotransmitters are produced from substrates derived from glycolytic pathways. Additionally, the enzymatic process of glycosylation is closely linked to neuronal glucose metabolism. In this post-translational modification, sugar chains (glycans) are covalently attached to proteins or lipids, which is essential for protein stability, folding, trafficking, and cell longevity. Neuronal activity and repair processes are interdependent on glucose-based energy metabolism.

Most of the energy required for normal neuronal function is derived from glycolysis and cellular respiration, as evidenced by the substantial production of ATP [61,63,64]. The proper progression of biochemical reactions associated with cellular activity, along with the restoration of homeostasis and repair processes, relies on the kinetics and concentrations of enzymes and cofactors, which can change with cellular aging and the accumulation of metabolic byproducts and calcium ions [60,61]. This results in a gradual decline in cellular metabolism coupled with inhibited ATP production, ultimately leading to apoptosis.

The intensity of glucose metabolism varies significantly across different regions of the brain, influencing their susceptibility to functional disorders and damage. Notably, cortical processes associated with neural networks—such as neurotransmission, synaptogenesis, synaptic pruning, and axonal myelination—are particularly energy-intensive [65]. Nerve cells consist of unstable biomaterials, which leads to gradual wear and tear, resulting in progressive degeneration and age-related loss of function. Maintaining neuronal functionality and metabolic efficiency relies on the continuous remodeling of cellular and subcellular membrane structures, including mitochondria [61].

Nicotinamide adenine dinucleotide (NAD) is a key metabolic coenzyme that significantly contributes to neuronal glucose-dependent bioenergetics, genome stability, adaptive stress responses, cellular homeostasis, and survival [66]. Physiological NAD levels and, consequently, glucose-dependent cellular processes are maintained by two salvage pathways, while the kynurenine pathway acts as a de novo pathway [66]. The contribution and effectiveness of both pathways in maintaining physiological NAD levels change with age, as well as due to disease processes and inflammation [66]. Under normal physiological conditions, the kynurenine pathway serves as a supplemental source of the coenzyme NAD [67]. Importantly, glutamine acts as the primary nitrogen donor in the final step of the de novo kynurenine pathway, converting nicotinic acid adenine dinucleotide to NAD [68].

The kynurenine pathway plays an essential role in modulating neuronal functions by regulating the activity of N-methyl-D-aspartate (NMDA) receptors [66]. It is a primary route for tryptophan metabolism, yielding neuroactive intermediates such as quinolinic acid, which acts as an excitotoxic NMDA receptor agonist, and kynurenic acid, which serves as a neuroprotective NMDA receptor antagonist. Quinolinic acid, a biosynthetic precursor of NAD, functions as both an NMDA receptor agonist and a neurotoxin [66,69,70]. The disruption of energy metabolism leads to neuronal depolarization and overactivation of NMDA receptors, causing an uncontrolled rise in intracellular calcium levels and initiating apoptosis [70]. Furthermore, dysregulation of the kynurenine pathway results in a significant reduction in SIRT1 activity in astrocytes and neurons. Generally, dysfunction of the kynurenine pathway can lead to either hyper- or hypofunction of active metabolites, profoundly impacting the functioning of the central nervous system and exacerbating neurodegeneration, neurological disorders, and psychiatric illnesses like depression and schizophrenia [71,72,73].

## 5. The Aging Brain

The brain’s functional structures consist of postmitotic, differentiated neurons that have permanently exited the cell cycle, meaning they can no longer divide [74]. Almost all neurons become terminally differentiated postmitotic cells early in development. Their longevity and proper functioning depend on highly stable molecular mechanisms [74]. Depending on their activity levels, the protein and lipid structures within neurons can degrade relatively quickly, typically within days or weeks [75]. Studies suggest that the half-life of neuronal mitochondria is approximately 2 to 4 weeks, with excessively used mitochondrial proteins eliminated by a process called mitophagy [75]. Therefore, to maintain proper brain function over the long term, lipoprotein-based cellular structures must be continuously rebuilt, and the byproducts of repair processes must be effectively removed. This includes the continuous division and fusion of mitochondria, as well as the repair of membrane channels and receptors, which rely on post-translational modifications of lipids and proteins through glycosylation.

Increasing age-related insufficiency of intracellular NAD salvage pathways and the disruption of the kynurenine pathway caused by chronic inflammatory processes do not allow for the supplementation of NAD deficiency and, at the same time, increase the production of excitotoxins such as quinolinic acid [76]. Elevated levels of quinolinic acid destabilize the cytoskeleton of astrocytes and blood vessel endothelial cells, which can degrade the blood–brain barrier [70]. Dysfunction of the kynurenine pathway leads to a rapid decline in NAD levels with a concomitant decrease in NAD-dependent SIRT1 activity, culminating in accelerated aging and ultimately neuronal death [61,77]. The accumulation of Aβ peptide in the brain occurs decades before the onset of Alzheimer’s symptoms. Aβ is produced from the membrane-bound amyloid β precursor protein (APP) through proteolytic cleavage by β-secretase 1 (BACE1) and γ-secretase [77]. Growing evidence indicates that the intracellular trafficking of APP to each secretase determines the level of Aβ production [77]. Importantly, cleavage by the α-secretase ADAM10 and γ-secretase does not produce amyloid β [77].

Aging, particularly the progressive decline in energy metabolism, significantly impacts neuronal function. Brain aging is the primary cause of behavioral changes such as low mood, anhedonia, reduced motivation, hypokinesia, and depression. It brings about multiple structural changes within the brain that reduce cognitive abilities, especially affecting learning and memory. Episodic memory is particularly vulnerable due to dysfunction in the hippocampus, as well as the surrounding perirhinal and entorhinal cortices, and the prefrontal cortex [78]. Dysregulated synaptic plasticity and reduced neurogenesis in the dentate gyrus of the hippocampus have been linked to deficits in episodic memory [79]. Additionally, reductions in gray matter and the loss of thin spines in layer 3 of the prefrontal cortex have been associated with declines in executive function [80].

Stress, which increases with age, is an inherent attribute of aging and especially of disturbing the energy balance of the brain. Chronically elevated cortisol levels can lead to glucocorticoid receptor resistance, inhibiting the activity of tryptophan 2,3-dioxygenase (TDO) in the liver. TDO catalyzes the first and rate-limiting step of tryptophan degradation, thereby impairing systemic tryptophan metabolism [73,81]. This results in an increased production of quinolinic acid, at the expense of NAD synthesis [76]. Such disorders lead to a significant decrease in SIRT1 activity in astrocytes and neurons. Thus, dysfunction of the kynurenine pathway can worsen the dysfunction of the glutamate–GABA–glutamine cycle, profoundly affecting the central nervous system. This exacerbates neurodegeneration, neurological disorders, and psychiatric illnesses, including depression and schizophrenia [71,72,73]. Increased concentrations of quinolinic acid in cerebrospinal fluid have been observed in several neurodegenerative diseases, including Alzheimer’s disease, Parkinson’s disease, multiple sclerosis, depression, epilepsy, and Huntington’s disease [82,83].

## 6. Chemical Imbalances in the Aging Brain

Neurons form intricate synaptic transmission networks that function under the influence of specific ions, neurotransmitters, neuromodulators, cytokines, and hormones. If neurons are lost, these connections cannot be restored [84]. Neuronal dysfunction and death are linked to the gradual degradation of multiple functional systems and neurotransmitters, particularly those in the glutamatergic, dopaminergic, serotonergic, GABAergic, histaminergic, adrenergic, and cholinergic systems within the cerebral cortex, brainstem, and basal ganglia [85]. The progressive decline in energy metabolism plays a critical role in neuronal aging, contributing to the failure of cellular processes and increased cell death [60,61]. Metabolic dysfunctions and related inflammatory processes throughout the body are at the root of the pathogenic process known as inflammaging [86,87]. Aging causes a shift in energy and metabolic resource allocation, shifting it toward combating chronic inflammation and supporting tissue regeneration and repair. This neuroimmune energy shift is regulated by the hypothalamic–pituitary–adrenal (HPA) axis [81].

### 6.1. Glutamate–GABA–Glutamine Cycle

The brain’s neocortex plays multiple roles in controlling human behavior, including sensory perception, working memory, and motor planning [8]. Its unique structure consists of repeating subunits known as macrocolumns, which generally have identical circuitry [8,28]. The core of each column is made up of glutamatergic excitatorypyramidal neurons, surrounded by GABAergic neurons that form a local inhibitory network. Glutamatergic excitatory pyramidal neurons account for approximately 70–80% of the total neuronal population, while the inhibitory GABAergic short axonal interneurons make up only 15–25% [28]. Such modular column organization is typical in the somatosensory and visual cortices, as well as the primary motor cortex. Distal dendritic segments are capable of generating impulses autonomously, allowing pyramidal neurons to learn new activation patterns independently of somatic action potentials. In contrast, synapses connecting to the proximal basal dendrites are more readily modified alongside global neuronal activity [8,81]. Calcium ions influxing through activated NMDA receptor channels play a critical role in long-term synaptic plasticity, which is essential for cortical memory processes [81].

Glutamate is the primary excitatory neurotransmitter in the human brain and is involved in numerous physiological functions such as cognition, memory, learning, neurodevelopment, cell migration, cell differentiation, and apoptosis [88,89]. Disrupted glutamate signaling is associated with the development of several neurological disorders [82]. In particular, excessive activation of N-methyl-D-aspartate (NMDA) receptors can lead to cascades of neuronal death [82]. Glutamate excitotoxicity primarily results from excessive calcium ion influx into neurons, which is essential for synaptic transmission, combined with a deficiency in the energy needed for calcium removal, leading to the death of particularly aging and energy-inefficient neurons. Glutamine is utilized to recycle the neurotransmitters glutamate and GABA, while excess glutamine supports neuronal energy metabolism through the tricarboxylic acid cycle by oxidizing acetyl-CoA derived from glycolysis [90].

The glutamine-dependent increase in ATP provides the energy needed to restore ionic balance at resting potential and to remove excess calcium ions. Impaired energy metabolism in astrocytes leads to reduced glutamate uptake due to insufficient ATP production. Under pathological conditions, astrocytes may fail to meet the energetic demands of glutamate uptake, causing intracellular glutamate accumulation. Reduced levels of glutamate and GABA in aging neurons result in an increased concentration of glutamine in the cerebrospinal fluid, significantly affecting microglial activity. The accumulation of glutamine in the brain can disrupt excitatory signaling and is associated with brain dysfunction or disease, manifesting as mental disorders, disorientation, personality changes, or even coma [90].

The glutamate–GABA–glutamine cycle is a major metabolic pathway in the brain, and its activity is directly proportional to cerebral oxidative glucose metabolism [90]. The rate of glutamate oxidation in astrocytes is strongly dependent on concentration. At low concentrations, most glutamate is converted to glutamine, while higher extracellular glutamate concentrations lead to increased oxidative metabolism, resulting in the production of reactive oxygen species (ROS) and α-ketoglutarate [90]. Glutamate acts as a carbon donor for the generation of α-ketoglutarate and serves as a nitrogen donor for the synthesis of glutathione (GSH) [91,92,93]. Reduced glutathione is the most abundant endogenous antioxidant, essential for maintaining redox homeostasis and protecting neurons from oxidative damage. It is particularly crucial for mitochondrial function and the maintenance of mitochondrial DNA, as well as for regulating cellular proliferation and apoptosis.

The glutamatergic and GABAergic systems are vital for maintaining the excitatory-inhibitory balance within the neocortex [94]. This balance is closely regulated by metabolic coupling between neurons, astrocytes, and microglia [90]. In particular, the glutamate–GABA–glutamine cycle serves as the primary metabolic complex linking neurotransmission with cellular metabolism. In this cycle, astrocytes synthesize and release large quantities of glutamine, which neurons subsequently uptake to produce the neurotransmitters glutamate and GABA [90]. Most synaptic glutamate is recycled from the synapse via astrocytic uptake, thus preventing overstimulation. Glutamate uptake is considered one of the most energy-intensive processes within the central nervous system [89,95]. A significant portion of the glutamate taken up by astrocytes is oxidatively metabolized to α-ketoglutarate, which then contributes to ATP production. In this way, oxidative glutamate metabolism leads to the production of massive amounts of ATP in astrocytes.

### 6.2. Glutathione

Glutamine is also a crucial precursor for the body’s conversion to glutamate, which supports glutathione synthesis [96]. The metabolism of glutathione has a significant impact on glutamate synaptic activity [97]. Disturbances in glutathione/glutamate metabolism can be triggered by inflammation, which is marked by increased glutathione production in the liver. In the brain, glutamine serves as an energy enhancer, facilitating increased ATP levels during periods of intense neuronal activity. Accumulating deficiencies in glucose metabolism and the glutamate–GABA–glutamine cycle can lead to ion pump dysfunction and prolonged depolarization, rendering neurons vulnerable to uncontrolled spontaneous firing and death due to energy deprivation or excitotoxicity. This uncontrolled neuronal activity results in excessive glutamate release and sustained activation of glutamate receptors, which exacerbates neuronal death, particularly in the hippocampus and cerebral cortex [98]. Dysregulation of glutathione is recognized as a contributing factor in the development of neurodegenerative diseases, such as Alzheimer’s disease, Parkinson’s disease, Huntington’s disease, and amyotrophic lateral sclerosis [99].

The synthesis of glutathione primarily occurs in astrocytes, from where it is then transported to neurons. In healthy, resting neurons, the reduced to oxidized glutathione ratio is around 100; however, this ratio can drop to 10 or less in cells exposed to oxidative stress [92,100,101]. A decreasing ratio indicates an imbalance often associated with aging and neurodegenerative diseases.

### 6.3. Glutamine

Glutamine is the most abundant amino acid in the human body, with its primary endogenous source being de novo synthesis in striated muscles [102,103]. The availability and metabolism of glutamine are directly related to skeletal muscle mass and activity, which account for up to 90% of total glutamine production in the body [103]. It is essential to note that motor behavior plays a significant role in glutamine metabolism, thus influencing brain function. Both skeletal muscles and the liver are the body’s main sites for glutamine synthesis, storage, and release, together accounting for about 70% of endogenous production in humans [103].

Glutamine, alongside glucose, is a vital energy substrate in the body, especially in tissues under metabolic stress and in rapidly dividing cells like lymphocytes and enterocytes [90,103]. Additionally, glutamine provides nitrogen necessary for the synthesis of purines, pyrimidines, and amino sugars. Consequently, a deficiency in glutamine can impair the function of various metabolic pathways and mechanisms reliant on its availability, leading to immune dysfunction and disturbances in the brain’s glutamatergic system [103]. Large amounts of glutamate, GABA, and glutamine are depleted through oxidative metabolism in both neurons and astrocytes. This deficiency can be compensated for by muscle-derived de novo synthesized glutamine, which can cross the blood–brain barrier (BBB) [104]. The BBB actively transports glutamine in both directions, regulating brain nitrogen levels, protecting against neurotoxicity, and maintaining astrocyte health. This barrier utilizes Na+-dependent glutamine transport across capillary endothelial cells, while also actively removing excess glutamine and ammonia from the brain into the bloodstream to prevent toxicity [104]. Early signs of cognitive and motor alterations may arise from a deregulation of the glutamate-glutamine cycle [105].

The availability and metabolism of glutamine in the body are closely tied to skeletal muscle mass and activity [103]. Muscle tissue is responsible for 90% of the body’s total glutamine production; thus, the role of skeletal muscle and its activity is fundamental in glutamine metabolism, significantly influencing brain function [103]. In physiological conditions, glutamine acts as an anaplerotic substrate in neurons, entering the tricarboxylic acid cycle as α-ketoglutarate. Alpha-ketoglutarate is a crucial intermediate for cellular energy production and amino acid metabolism, significantly influencing the rate of the body’s citric acid cycle [106]. It acts as a nitrogen scavenger and a source of glutamate and glutamine, which stimulate protein synthesis and inhibit protein degradation in muscle [106]. In the brain, alpha-ketoglutarate serves as a precursor for key neurotransmitters, including glutamate, GABA, and acetylcholine.

In inflammatory states, the liver shifts from being a glutamine producer to becoming its primary consumer. During inflammation, the brain relies exclusively on de novo glutamine production in skeletal muscle to sustain gluconeogenesis [103]. In older adults, reduced physical activity, coupled with chronic inflammation, can lead to muscle atrophy and decreased glutamate production [103,105]. This situation further escalates inflammatory processes and contributes to the progressive deterioration of nervous and mental functions.

Glutamine deficiency occurs early in the aging brain. Factors such as hypoglycemia, insulin resistance, and age-related NAD deficiency contribute to excessive glutamine oxidation in brain cells. This leads to reduced glutamine synthesis in astrocytes, which directly impairs neuronal GABA signaling [107]. As a result, some glutamatergic neurons may become hyperactive in the early stages of Alzheimer’s disease, potentially causing seizures and accelerating disease progression. A significant reduction in hippocampal glutamine synthesis is also observed in patients with temporal lobe epilepsy, which results in excessive intracellular glutamate accumulation in astrocytes. This accumulation reduces synaptic glutamate uptake, leading to neuronal hyperexcitability and seizures [108].

As individuals age, glutamine deficiency can impair immune function, which subsequently disrupts the brain’s glutamatergic system. Particularly, during inflammatory states, brain glutamine needs are completely dependent on muscle resources [103]. Thus, in older adults, the accumulation of chronic inflammation and metabolic disturbances, combined with systemic glutamate deficiency, leads to muscle atrophy and sarcopenia [103]. This, in turn, further exacerbates inflammatory responses, leading to progressive neurodegeneration and deterioration of neuronal and mental functions.

### 6.4. Catecholamines

Catecholamines include norepinephrine (noradrenaline), epinephrine (adrenaline), and dopamine. Norepinephrine plays a crucial role in regulating arousal, alertness, attention, and memory, forming a key element of the eustress response. By maintaining wakefulness, norepinephrine enhances the detection of sensory stimuli, improves concentration, sustains attention, and supports memory formation and recall. Loss of these functions is common in neurodegenerative diseases, contributing to cognitive and behavioral deficits [109]. Almost all norepinephrine in the brain is produced in the locus coeruleus, a small brainstem nucleus that sends signals to nearly all brain regions, including the prefrontal cortex, hippocampus, and amygdala. Notably, norepinephrine is the only neurotransmitter synthesized in the synaptic vesicles of locus coeruleus neurons [110]. Noradrenergic neurons in this area are also among the first to develop α-synuclein inclusions [110]. As a result, many early symptoms of brain neurodegeneration, such as sleep disturbances, anxiety, and depression, have been linked to locus coeruleus dysfunction [110].

During states of high alertness, noradrenaline released from the locus coeruleus acts on astrocytes, where it works together with glutamate to activate intracellular inositol trisphosphate (IP3) messenger, facilitating calcium release from the endoplasmic reticulum [111]. In response, the activated astrocytes release gliotransmitters, including D-serine, which coactivates NMDA receptors in the noradrenaline varicosity, modulating synaptic plasticity and information transfer [112].

The cellular population of the locus coeruleus consists of over 95% noradrenergic neurons [109]. The distribution of locus coeruleus projections is uneven throughout the neocortex, with axonal varicosities serving as sites for synaptic vesicle storage and neurotransmitter release. These projections primarily target key motor structures, such as the cerebellum, inferior olive, and motor cortex, as well as the olfactory bulb, frontal cortex, visual cortex, thalamus, midbrain, and spinal cord. The noradrenergic system of the locus coeruleus influences several fundamental behavioral processes and executive functions that depend on the prefrontal cortex, including synaptic plasticity and memory formation. Activity in the locus coeruleus during sleep features periodic switches between activity and silence, playing a crucial role in memory consolidation. The intense neural activity within limbic circuits during sleep enhances synaptic plasticity [110].

Noradrenergic neurons in the locus coeruleus are particularly vulnerable to degeneration during aging [110]. The age-related decline in neuroadrenergic activity leads to a gradual loss of neuromelanin’s protective functions, exacerbating degenerative processes [109]. Early symptoms of noradrenergic signaling deficiency are often associated with difficulties in maintaining attention and excessive motor activity. Dysfunction in the locus coeruleus is strongly linked to numerous neuropsychiatric and neurological diseases due to its role in maintaining homeostasis and modulating neuroinflammation [113]. Therefore, current antidegenerative therapies target the locus coeruleus-noradrenaline circuits, including the suprachiasmatic nucleus and dorsomedial hypothalamus [110].

Adrenergic neurons, primarily located in the medulla oblongata, regulate key functions such as cardiovascular control, respiration, and stress responses. These neurons contain the enzyme phenylethanolamine N-methyltransferase (PNMT), which catalyzes the final step in catecholamine biosynthesis by converting noradrenaline into adrenaline. In this manner, they send signals to the hypothalamus and spinal cord, serving as central modulators. The hypothalamus executes these functions by controlling the pituitary gland, thereby regulating most endocrine responses [114].

Age-related declines in noradrenergic activity impair the regulation of neural plasticity, thus contributing to memory and learning difficulties in older adults [110]. Dysfunction of the locus coeruleus–noradrenaline system is associated with various stress-related neuropsychiatric disorders and neurological diseases [113]. Significant neuronal loss in the locus coeruleus is considered one of the early features of Alzheimer’s disease and dementia in Parkinson’s disease [115]. The combined deficits in cholinergic and dopaminergic systems appear to contribute to cognitive impairment in Parkinson’s disease, supporting the dual syndrome hypothesis [115]. When comparing cases of Parkinson’s and Alzheimer’s diseases, the loss of cholinergic signaling is either comparable to or more severe in Parkinson’s disease. However, this effect is particularly pronounced in patients with both Parkinson’s disease and dementia [115]. Consequently, patients with dementia tend to respond better to anticholinesterase drugs, such as rivastigmine and galantamine, which are used for the symptomatic treatment of mild to moderate dementia in both Alzheimer’s and Parkinson’s diseases [115]. The decrease in acetylcholine levels in the cerebral cortex observed in dementia may be linked to cell death within the nucleus basalis of Meynert (nbM) [115]. In Alzheimer’s disease, there is a loss of 8% to 87% of magnocellular neurons in the nbM compared to the control group, while in Parkinson’s disease, this loss can reach up to 80% [115].

Dopamine, the third catecholamine, plays a crucial role in regulating various aspects of human life, including motor behavior, memory, and learning, as well as the perception and processing of emotional and motivational stimuli [116]. Dopamine is a key reward neurotransmitter responsible for the feeling of pleasure. The mesolimbic dopaminergic system connects the ventral tegmental area to the nucleus accumbens, amygdala, and hippocampus, significantly influencing reward-motivated learning and behaviors [117,118]. The nucleus accumbens is a key node in the mesolimbic pathway that increases dopamine levels in the ventral tegmental area, inducing positive reinforcement, which is essential for engaging in physically demanding activities [119]. Adenosine receptors are primarily expressed on GABAergic medium spiny neurons in the striatum and nucleus pallidus, co-expressing dopamine and enkephalin receptors, which link motor behaviors with motivation and reward [120]. Beyond motor control, these pathways also regulate cognitive, limbic, and reward functions, playing a critical role in the development of cortical circuits [120].

In the prefrontal cortex, dopamine facilitates the selection and successful execution of behaviors aimed at achieving specific goals [118]. Dysfunctional dopamine signaling is a causal factor in many symptoms associated with neurodegenerative and neuropsychiatric disorders, including autism, attention deficit hyperactivity disorder (ADHD), and schizophrenia [121]. A selective loss of dopaminergic neurons in the substantia nigra pars compacta is a hallmark of Parkinson’s disease, which presents motor symptoms such as hypokinesia, tremors, rigidity, and postural imbalance [122]. The dorsal striatum directly contributes to decision-making, particularly in action selection and initiation, by integrating sensorimotor, cognitive, and motivational/emotional information within specialized corticosteroid circuits [123]. It plays a vital role in controlling motor behavior, especially locomotion, by providing egocentric navigation strategies [124].

### 6.5. Acetylcholine

In the central nervous system, acetylcholine is the excitatory neurotransmitter, predominantly found in the basal forebrain. Acetylcholine is synthesized solely in the cytoplasm of neurons from choline and acetyl-CoA, with ATP being an essential component of this process [125]. It functions as a neuromodulator critical for arousal, memory, and learning. The cholinergic neurons have extensive projections that provide the primary input to the cerebral cortex and hippocampus [126].

The nucleus basalis of Meynert (nbM) is critical for cholinergic activation of the cerebral cortex, as it coordinates the activities of the glutamatergic and GABAergic systems during learning and memory [127]. The nbM is involved in cognitive processes, including attention, memory, perception, and arousal [115,126]. It receives synaptic input from the ventral and dorsal striatum, hypothalamus, amygdala, and brainstem tegmentum, providing widespread cholinergic input to the cerebral cortex, hippocampus, thalamus, and amygdala [127]. Local cholinergic neurons primarily reside in the striatum, nucleus accumbens, olfactory tubercle, hippocampus, and layers II–V of the cerebral cortex [126].

This extensive control system is highly sensitive to damage, overload, and stress. The degradation of the cholinergic system can lead to observable changes in human motor behavior, making it useful for diagnostic purposes. The most vulnerable cholinergic centers are particularly susceptible to aging and damage. Various pathological conditions, such as Alzheimer’s disease, Parkinson’s disease, schizophrenia, autism, and attention deficit disorder, can develop as a result [126]. The combined neurodegeneration of the dopaminergic and cholinergic systems is believed to explain cognitive impairment and dementia symptoms in neurodegenerative diseases. Significant neuronal loss in the nucleus basalis of Meynert is considered an early feature of dementia in Alzheimer’s and Parkinson’s diseases [115]. In these conditions, the loss of cholinergic neurons in the nucleus basalis of Meynert can reach up to 80% [115].

Acetylcholine, also a key excitatory neurotransmitter in the peripheral nervous system, is responsible for skeletal muscle contraction. The activity of human skeletal muscles is controlled by the largest nerve cells, known as motor neurons, which are distinguished by their long axon fibers that can reach over 1 m in length and a highly developed axonal tree. A single motor neuron can control several hundred to nearly 2000 muscle fibers via cholinergic synapses. At the neuromuscular junction, released acetylcholine is quickly degraded by the enzyme acetylcholinesterase into choline and acetate, thereby terminating cholinergic synaptic transmission. Choline is then reabsorbed by the presynaptic neuron, where it combines with acetyl-CoA to form acetylcholine via the action of choline acetyltransferase [125]. Choline plays a direct role as a precursor to acetylcholine and phospholipids, serving as a key building block for structural lipids like phosphatidylcholine and sphingomyelin, which are vital for maintaining the structure and function of cell membranes [128].Choline deficiency can lead to muscle damage, cognitive deficits, and fatty liver [128]. These adverse effects are worsened by the age-related decline in NAD levels, leading to a significant reduction in neuronal glycolysis and oxidative phosphorylation [60,61,129,130]. Thisdeficiency contributes to motor neuron death and a progressive loss of muscle mass (sarcopenia) in older adults, creating a vicious cycle of inflammation.

Choline also supports the synthesis of very low-density lipoprotein (VLDL) particles, which play a vital role in transporting lipids from the liver and help prevent fatty liver disease [128]. Consequently, choline protects liver-regulated glucose metabolism, and choline supplementation may help prevent age-related memory decline and protect the brain from neuropathological changes associated with Alzheimer’s disease and neurological damage linked to epilepsy and hereditary disorders such as Down syndrome and Rett syndrome [128]. Therefore, symptomatic treatment with anticholinesterase drugs is recommended for individuals experiencing mild to moderate dementia [115].

The α7 nicotinic acetylcholine receptors found on macrophages, dendritic cells, and lymphocytes play a crucial role in modulating inflammation. This occurs primarily through their interaction with acetylcholine released by the vagus nerve [131,132]. When acetylcholine binds to these receptors on immune cells, it triggers the intracellular JAK2-STAT3 signaling pathway, which inhibits the production of pro-inflammatory cytokines such as TNF-α, IL-1β, and IL-6, thus reducing the inflammatory response. Activation of the vagus nerve leads to decreased production of pro-inflammatory cytokines and suppression of an exaggerated immune response. Currently, α7 receptor agonists are being tested for the treatment of inflammatory conditions as well as neuropsychiatric disorders [132]. Transcutaneous vagus nerve stimulation has shown positive effects on psychiatric and neurological disorders such as depression, epilepsy, and cognitive dysfunction. Stimulation can reduce inflammation-related depression and anxiety and may alleviate chronic pain, including fibromyalgia and headaches and these effects are mediated, at least in part, by the activation of the locus coeruleus [110].

### 6.6. Serotonin

Nearly all pyramidal neurons in the human prefrontal cortex, as well as approximately 30% of GABAergic interneurons, express the serotonin receptor [133]. Activation of serotonin in the prefrontal cortex inhibits neurons in the dorsal raphe nucleus, which is the brain’s primary serotonergic nucleus and serves as the main projection point for serotonin to the forebrain. This negative feedback loop is crucial for regulating mood, sleep–wake cycles, reward, aversion, and pain [133]. Deficits in the serotonergic system can lead to various pathological conditions, particularly major depression, post-traumatic stress disorder, schizophrenia, attention deficit hyperactivity disorder (ADHD), mood disorders, and autism [134].

### 6.7. Neuropeptides

Hypothalamic neuropeptides are significantly larger molecules than classical neurotransmitters. Their synthesis and transport require substantially more energy [135]. Unlike classical neurotransmitters, their release is not confined to synapses, although portions may be co-secreted with these neurotransmitters. Due to their extended half-life, orexins can diffuse over long distances, producing paracrine and endocrine effects by activating G protein-coupled receptors (GPCRs). Their effects develop slowly but tend to be sustained for longer periods [135]. A deficiency in orexin-producing cells leads to narcolepsy type 1, which is characterized by excessive daytime sleepiness, loss of muscle tone, sleep paralysis, and hallucinations [135].

The hypocretin/orexin system represents a highly complex neuropeptide network in the brain that regulates various physiological and behavioral processes [135]. In the human brain, there are approximately 50,000 to 80,000 orexin-producing neurons, predominantly located in the perifornical area and lateral hypothalamus. However, their axon terminals extend to distant regions, and their receptors are distributed throughout the entire brain [135]. Orexins act as master regulators of vigilance and stress responses and receive significant input from neurons in the circadian system, with the suprachiasmatic nucleus (SCN) being the most important center. In addition to regulating arousal, orexins influence appetite, feeding behavior, reward, and emotions [135].

### 6.8. Monoamine Oxidases

Monoamine oxidases (MAO) are a family of enzymes located in the outer mitochondrial membrane of neurons and astrocytes that catalyze the breakdown of neurotransmitters. They play a key role in regulating levels of serotonin, dopamine, and norepinephrine [136]. The isoform MAO-A is primarily found in dopaminergic, noradrenergic, and adrenergic neurons, while MAO-B is predominantly located in astrocytes. The activity of both isoforms helps maintain the neurochemical balance that affects mood. Increased MAO-B activity enhances GABA production, leading to excessive tonic inhibition, which impacts neuronal excitability and synaptic function. Furthermore, metabolism through MAO-B generates reactive oxygen species (ROS), including hydrogen peroxide, exacerbating oxidative stress and neuroinflammation. Overexpression of MAO-B and depletion of dopamine are considered pathological factors in neurodegenerative diseases, including Parkinson’s and Alzheimer’s disease [137]. Conversely, MAO-B deficiency may lead to a hyperdopaminergic state and behavioral disinhibition in schizophrenia [138]. Importantly, skeletal muscle also exhibits significant monoamine oxidase activity, primarily MAO-B, and serves as the main source of this enzyme in healthy humans. However, under chronic stress conditions, high glucocorticoid levels and increased MAO-A expression can promote protein degradation and inhibit protein synthesis, contributing to muscle atrophy and mitochondrial damage through heightened production of reactive oxygen species [138].

### 6.9. Calcium

N-methyl-D-aspartate (NMDA) glutamate receptors are vital for excitatory synaptic transmission in the central nervous system, playing a significant role in synaptic plasticity [139]. In the adult brain, over 80% of neurons and more than 90% of synapses release glutamate [140]. NMDA receptors function as key calcium ion channels essential for learning and memory [139]. Voltage-gated sodium-calcium channels are crucial for the activity of cells with variable activity levels. They trigger action potentials in neurons and muscle cells, coupling plasma membrane depolarization to intracellular events such as secretion, contraction, synaptic transmission, and gene expression [141]. The sodium-calcium exchanger serves as a transporter, removing intracellular Ca^2+^ ions by using the electrochemical gradient to exchange them for Na^+^ ions.

In astrocytes, calcium in the cytosol acts as a universal messenger, responding to various stimuli and triggering a range of cellular responses. Astrocytic calcium levels change dynamically with sleep and wake states [142]. During sleep, activity in the locus coeruleus is low, resulting in suppressed or absent astrocyte calcium responses. In contrast, pronounced astrocyte calcium responses, driven by norepinephrine released from the locus coeruleus, occur during locomotion [143]. Under conditions of high arousal, glutamate transfer to active synapses enhances local norepinephrine release from locus coeruleus axons [144]. Large-scale calcium waves in the aroused brain are primarily inositol trisphosphate IP3-dependent, primarily evoked by sensory input, and contribute to reliable sensory transmission. Localized calcium spikes appear to be IP3-independent and are associated with decreased extracellular potassium (K^+^) levels, hyperpolarization of neurons, and suppression of sensory transmission. In the aging brain, several secondary complications, such as impaired calcium signaling and stem cell depletion, lead to dysfunction in neural networks [66]. Calcium and phosphate signaling play fundamental roles in these processes.

Calcium ions enter nerve and muscle cells through voltage-gated calcium channels and activate intracellular ryanodine calcium channels located in the endoplasmic and sarcoplasmic reticulum (ER/SR) [145]. The release of intracellular calcium into the cytoplasm triggers essential processes, including mitochondrial dynamics and activity [146]. Specifically, calcium ion binding to mitochondrial membranes limits the proton current involved in ATP synthesis. Hence, the activity of mitochondria and the endoplasmic reticulum, along with their interactions, is modulated by calcium signaling, and deficiencies in this interaction can lead to apoptosis. The locus coeruleus, which has extensive projections throughout the brain and supplies norepinephrine, is particularly active during wakefulness. During sleep, locus coeruleus activity decreases, leading to suppressed or absent astrocyte calcium responses. In contrast, notable astrocyte calcium responses occur during locomotion, driven by norepinephrine from the locus coeruleus [147]. Under high arousal conditions, glutamate transfer to active synapses enhances local norepinephrine release from locus coeruleus axons [144].

## 7. Gut–Brain Axis Dysfunction in the Aged

The gut–brain axis is a bidirectional communication network linking the enteric nervous system with the brain. It enables constant, bidirectional signaling via neural, immune, and hormonal pathways, profoundly influencing mood, digestion, and immunity [147]. The gut microbiota plays a crucial role in this axis, with gut health directly affecting brain function. Gut microbiota can influence the brain, neurochemistry, physiology, and behavior [49]. The microbiota plays a role in this process, in part by generating short-chain fatty acids, which help regulate microglial homeostasis and are essential for their maturation and function [148]. Human studies have shown a link between irritable bowel syndrome, chronic inflammation, and depression. In older adults, dysbiosis can disrupt the intestinal barrier, allowing endotoxins, such as lipopolysaccharides, to enter the bloodstream, which leads to chronic inflammation. Infections and stress can cause dysbiosis, exacerbating microglial activation and dysfunction in schizophrenia [149]. Chronic, mild inflammation can damage the liver, promote insulin resistance, and contribute to the development of metabolic syndrome. In inflammatory conditions such as endotoxemia, the liver stops being the primary producer of glutamine and becomes its primary consumer. This metabolic reprogramming is driven by a significant increase in hepatic glutamine uptake, which depletes its availability in the brain. This mechanism can result in progressive dysfunction of the glutamine/glutamate-GABA cycle in cortical structures, leading to memory loss and learning difficulties.

Changes in the composition of intestinal microbiota, known as dysbiosis, have been linked to various human diseases, including cardiovascular, kidney, and skin diseases, as well as neurodegeneration [150]. The presence of endotoxin in the blood, along with the inflammation it triggers, may contribute to the compromise of the blood–brain barrier, potentially allowing toxins to penetrate the central nervous system [150]. Metabolic endotoxemia is characterized by a two- to three-fold increase in blood endotoxin concentration and mild inflammation. In obese individuals, metabolic endotoxemia is often associated with metabolic syndrome, which includes dyslipidemia, hypertension, and insulin resistance. Metabolic syndrome is a significant contributor to cardiovascular disease and is linked to neurological disorders [150].

Infections and stress can result in dysbiosis, which may worsen microglial activation and dysfunction in schizophrenia [149]. Chronic, mild inflammation can damage the liver, promote insulin resistance, and contribute to the development of metabolic syndrome. During inflammatory conditions like endotoxemia, the liver shifts from being the primary producer of glutamine to becoming its main consumer. This metabolic reprogramming is driven by a significant increase in hepatic glutamine uptake, which depletes its availability in the brain. This pathogenic mechanism leads to progressive dysfunction in the glutamine/glutamate-GABA cycle within cortical structures, resulting in memory loss and learning difficulties. Changes in the composition of the intestinal microbiota, termed dysbiosis, have been linked to several human diseases, including cardiovascular, kidney, and skin diseases, as well as neurodegeneration [150]. The presence of endotoxin in the blood, along with the inflammation it induces, may contribute to a weakening of the blood–brain barrier, potentially allowing endotoxins to penetrate the central nervous system [150].

Metabolic endotoxemia is characterized by a two- to three-fold increase in blood endotoxin concentration and mild inflammation. In obese individuals, metabolic endotoxemia often coexists with metabolic syndrome, which includes dyslipidemia, hypertension, and insulin resistance. Metabolic syndrome is a significant contributor to cardiovascular disease and is associated with neurological disorders [150]. Endotoxin exposure can lead to inflammation in the nervous system, resulting in elevated levels of cytokines and increased cortisol. Individuals exposed to endotoxin may experience depressive symptoms, including low mood, sadness, irritability, fatigue, anhedonia, and loss of appetite [150]. Additional effects include sleep disturbances, decreased appetite, and impaired long-term memory, along with microglial activation and inflammation, as evidenced by the presence of pro-inflammatory cytokines like TNFα and IL-6. Peripheral administration of endotoxin can adversely affect synaptic plasticity, as well as hippocampal-dependent learning and memory.

## 8. Neuroglia Interactions in the Aging Brain

The interaction between astrocytes, neurons, and microglia is crucial for brain energy metabolism, maintaining neuronal homeostasis, and supporting synaptic function [151]. Neurons require high energy levels for synaptic activity and neurotransmitter synthesis, primarily generated through oxidative phosphorylation and the tricarboxylic acid cycle. Astrocytes mainly rely on anaerobic glycolysis and provide neurons with lactate and glutathione, which are essential for neuronal function and protection against oxidative stress. Microglia, as resident immune cells, can switch between oxidative phosphorylation and glycolysis, depending on their functional mode and activation state [151]. In their inflammatory mode, microglia phagocytose toxic aggregates, including β-amyloid (Aβ) plaques, dying cells, and debris, to restore tissue homeostasis [32]. This process involves recognizing externalized phosphatidylserine, a potent “eat me” signal in the nervous system during development and injury. The externalization of phosphatidylserine can be triggered by excessive calcium ion influx into axon terminals, initiating synaptic apoptosis.

**Figure 3:**
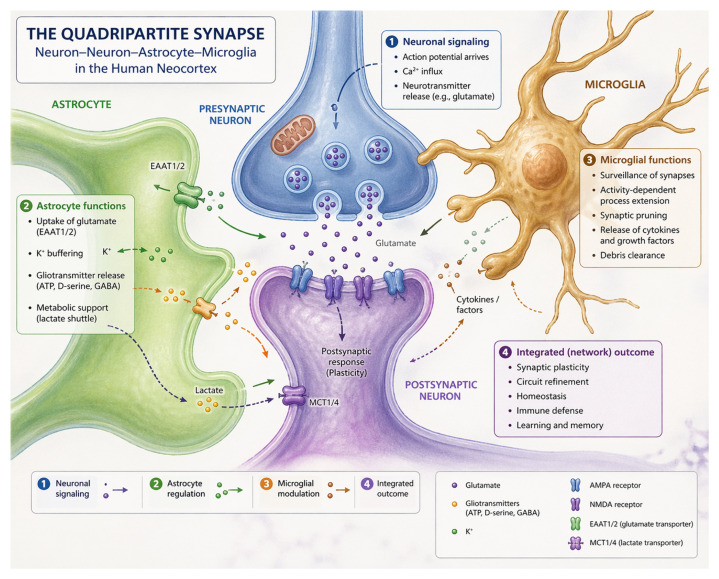
The quadruple synapse model suggests that the transmission of synaptic signals is regulated not only by neurons but also by astrocytes and microglia. Astrocytes play a crucial role in maintaining homeostasis and reabsorbing neurotransmitters, while microglia are involved in eliminating inactive synapses, dead neurons, and their debris.

Microglia make up approximately 10% of the cells in the nervous system and are the most abundant mononuclear phagocytes in the brain. Their activity and function are tightly regulated by the surrounding microenvironment, which is continuously influenced by mobile microglial processes that enable contact with neighboring neurons, astrocytes, and blood vessels (Figure 3). Microglia specifically monitor the functional state of synapses [49]. The level and manner of microglial activity depend on the neural network’s activity, allowing them to respond to changes in local neuronal activity and contribute to the maintenance of synaptic connections [49]. Microglia can influence synaptic strength and plasticity through the release of pro-inflammatory cytokines, reactive oxygen species (ROS), nitrogen species (NO), and neurotrophic factors. Microglia possess several receptors for neurotransmitters and neuropeptides released by neurons, facilitating communication between the two cell types. Receptors for neuropeptides such as substance P and bradykinin can promote the transition of microglia to an inflammatory mode, thus exacerbating inflammation. Additionally, GABA receptors, along with adrenergic, dopaminergic, and cholinergic receptors, modulate the microglial inflammatory response.

Microglia are a fundamental component of the brain’s intrinsic immune system. As a major source of pro-inflammatory cytokines, they play a crucial role in neuroinflammation and phagocytosis. To function effectively, microglia express immune receptors that regulate the intensity and duration of their activation. These receptors include immunoglobulin superfamily molecules (Ig-SF), which transmit activating or inhibitory signals through tyrosine kinase and tyrosine phosphatase pathways, respectively [49]. In a healthy adult brain, neuronal death can occur alongside plastic changes, but neurodegenerative processes tend to increase with age. Dead cells are quickly removed by phagocytes, helping to maintain the integrity of the cell membrane [152]. However, if apoptotic cells are not cleared away, they may become necrotic, leading to cell membrane breakdown and the leakage of intracellular molecules. This leakage triggers an inflammatory response in surrounding glial cells, resulting in inflammation and tissue damage [152]. Additionally, the accumulation of cell debris from axons and myelin can create physical or molecular barriers to axon growth during both development and disease. In the aging and diseased brain, the phagocytic activity of microglia can decline, causing prolonged dyshomeostasis and increasing the susceptibility of pyramidal neurons to degeneration [32].

The activity of AMPA and NMDA glutamate receptors, as well as mGluR2 metabotropic glutamate receptors, connects microglial functions to energy metabolism and the glutamate–GABA–glutamine cycle [49]. Deficiencies in synapse formation, glutamate signaling abnormalities, and impaired development of neuronal circuits can lead to serious neurological conditions, including epilepsy, Alzheimer’s disease, Parkinson’s disease, schizophrenia, and depression [93]. In these pathological conditions, neurodegeneration often extends beyond the hippocampus to include surrounding structures [98]. Neuronal damage localized outside the hippocampus is associated with amnesia. Glutamate activates microglial NMDA receptors, which in turn activate NADPH oxidase and release reactive oxygen species [49]. Conversely, ATP-stimulated microglia release brain-derived neurotrophic factor (BDNF), which activates tropomyosin receptor kinase (Trk) in spinal cord neurons and disrupts chloride homeostasis. Consequently, inhibitory signals mediated by GABA and glycine receptors are transformed into excitatory signals, leading to neuronal hyperexcitability and neuropathic pain [49].

## 9. Neurodegenerative Diseases and Disorders

### 9.1. Pathogenic Impact of Sleep Disorders

The body has a limited innate metabolic capacity, which means that essential processes occur in alternating cycles of wakefulness and sleep. While these two phases significantly differ in motor activity levels, the brain’s glucose-based energy metabolism decreases slightly during sleep [153]. During sleep, energy metabolism is directed toward neural processes rather than movement. These processes primarily involve neural repair, removal of waste metabolites, restoration of homeostasis, reorganization of synaptic connections, and adult neurogenesis, all of which are vital for learning and memory. Circadian rhythms, especially sleep, play a crucial role in these processes [154,155]. During sleep, the widening of intercellular spaces in the brain facilitates the removal of metabolic waste products, such as beta-amyloid, from the central nervous system. Specifically, intercellular spaces dilate during sleep, allowing glymphatic cells to efficiently remove these waste products [154,155,156].

Sleep disorders observed in older adults can impair this process, making it challenging to maintain a stable environment necessary for optimal neuronal function, nutrient distribution, and waste removal [153]. Sleep deficiency leads to several pathological processes, including inflammation, protein waste accumulation, and excitotoxicity, which can rapidly deteriorate brain health. Chronic sleep loss is associated with astrocytic phagocytosis of synaptic elements, resulting in the activation of microglia [157]. Even a single night of sleep deprivation can lead to the accumulation of amyloid-beta (A*β*) in the brain tissue of healthy individuals. Alzheimer’s disease is linked with both poor sleep and increased A*β* deposition in the brain, with a reported 95% reduction in A*β* clearance during wakefulness and activation of this process during deep sleep, which causes a 60% increase in extracellular space in the parenchyma [157].

Aquaporin 4 water channels on the endfeet of astrocytes optimize brain fluid movement and waste clearance, showing a significant increase in perivascular spaces during deep sleep [157]. Astrocyte contraction during sleep is regulated by the sodium–potassium pump (Na+/K+ ATPase). Astrocytes help clear excessive extracellular potassium due to high neuronal activity, creating osmotic gradients that result in water influx through aquaporins and subsequent astrocytic dilation. Increased neuronal activity, in which astrocytes facilitate the removal of excess potassium, is accompanied by an inward flow of chloride ions into the astrocytes, leading to significant swelling [142].

Sleep dysfunction triggers astrocyte reactivity and disrupts aquaporin-4 polarization, impairing cerebrospinal fluid flow and reducing clearance of α-synuclein and other waste products. These conditions can increase dopaminergic neuron death, exacerbating the motor deficits characteristic of Parkinson’s disease [158]. The severity of neurodegenerative processes is associated with sleep disorders stemming from chronic stress caused by glucose metabolism deficits [155]. Neurons are particularly vulnerable to low energy levels and oxidative stress. Insufficient sleep gradually increases oxidative stress and impairs mitochondrial function [159]. Both oxidative stress and mitochondrial dysfunction are critical factors in the pathology of neurodegenerative disorders. Prolonged sleep disturbances ultimately lead to an imbalance of normal cellular processes and an overproduction of reactive oxygen species [159]. These harmful compounds can damage lipids, proteins, and even DNA, leading to dysfunction in intracellular mitochondria, which are responsible for energy production. This dysfunction reduces ATP production, further compromising energy supply to the brain.

### 9.2. Neurodegenerative Diseases

Neurodegenerative diseases are characterized by the progressive deterioration of brain structure and function [160]. These diseases selectively affect various neuronal populations, leading to symptoms that are predominantly motor in conditions such as amyotrophic lateral sclerosis (ALS), Parkinson’s disease, and Huntington’s disease, or cognitive in disorders like Alzheimer’s disease and frontotemporal dementia [161]. Additionally, neurodegenerative diseases are associated with metabolic changes, which may include weight gain, weight loss, loss of fat mass, and altered feeding behavior [161]. Disordered energy homeostasis is a significant precursor in the progression of these diseases, suggesting that identifying pathways that lead to impaired energy balance may provide valuable therapeutic targets [61,70,161].

Signals from both peripheral and central sources are integrated in the hypothalamus, a major hub for energy balance control [161]. Neurodegenerative diseases typically involve the degeneration of specific neuronal populations, leading to characteristic symptoms [161]. These diseases may also feature additional symptoms and signs, including weight loss, endocrine disturbances, altered mood, and behavioral changes. Interestingly, many of these features could result from damage to the hypothalamus, which serves as an integration center between the brain and its environment. Energy balance is also regulated by the solitary tract nucleus, raphe nuclei, and ventral tegmental area. Huntington’s disease is neuropathologically classified based on neuronal loss and atrophy of the striatum within the basal ganglia. The motor symptoms of this disease are associated with the progressive dysfunction and death of medium-sized projection neurons in the striatum. Patients with frontotemporal dementia often show hypothalamic atrophy, primarily in the posterior region, including the lateral hypothalamic area nucleus and the dorsomedial nucleus [162].

Amyotrophic lateral sclerosis is initially characterized by muscle twitching, cramping, or difficulties with speech and swallowing, followed by progressive muscle weakness and atrophy, ultimately resulting in death, typically due to respiratory failure [163]. The symptoms of ALS are generally attributed to the combined degeneration of upper motor and lower motor neurons in the brain and spinal cord [163]. Approximately 50% of ALS patients exhibit hyperlipidemia and glucose intolerance, with or without insulin resistance [164,165,166]. In the presymptomatic stage, ALS patients often have a higher daily energy intake to compensate for increased energy expenditure, which may heighten the risk of hyperlipidemia and insulin resistance [167].

The onset of illness and cognitive symptoms in schizophrenia is linked to increased microglial pruning [149]. Synaptic pruning is closely regulated by complement component 4 (C4), which plays a crucial role in marking targets for removal, facilitating opsonization, and acting as an inflammatory mediator [149]. Both astrocytes and microglia serve as brain phagocytes, clearing away dead neurons and cellular debris, including synapses and axons. Thus, both cell types are essential in the process of synaptic pruning. Additionally, astrocytes also scavenge extracellular protein aggregates, such as β-amyloid (Aβ) and α-synuclein [152]. Neuronal activity and the anatomical connections between synapses and glial cells may determine whether synapses are engulfed by astrocytes or microglia. Importantly, sleep disturbances or acute sleep deprivation selectively enhance astrocyte phagocytosis in the cerebral cortex [152].

Programmed cell death is one of the most critical mechanisms influencing the structure and function of the brain [84]. Biochemical apoptotic cascades can be activated locally within synapses and dendrites or may involve entire neurons. During learning and memory processes, local apoptotic mechanisms allow for selective modification of the synaptic network through synaptic pruning, without requiring the elimination of entire neurons. However, in the aging brain, deficits in energy metabolism can lead to the accumulation of harmful factors, including the loss of membrane phospholipid asymmetry, membrane depolarization, mitochondrial dysfunction, calcium overload, and an increase in reactive oxygen species, which can result in substantial neuronal cell death. The activation of glutamate receptors primarily initiates apoptosis, and significant neuronal loss is a common symptom of neurodegenerative diseases.

### 9.3. Proteinopathies in the Aging Brain

Neuronal energy metabolism relies on the proper functioning of mitochondria, and mitochondrial dysfunction is associated with various brain disorders. The brain employs autophagy to eliminate damaged or redundant mitochondria, thereby maintaining neuronal health and homeostasis. An accumulation of unprocessed mitochondria elevates oxidative stress and further disrupts cellular energy metabolism. Deficiencies in mitophagy are linked to conditions such as the accumulation of beta-amyloid and tau proteins [159]. At physiological concentrations, amyloid beta is essential for synaptic plasticity in learning and memory. As a signaling molecule, it regulates synaptic strength, glutamatergic neurotransmission, cholesterol transport, and even offers protection against oxidative stress [168]. In healthy neurons, the sialylation of amyloid precursor protein (APP) imparts essential properties for its function in synaptic transmission [169].

Apolipoprotein E (apoE) utilizes cholesterol derived from astrocytes to transport neuronal APP into and out of lipid clusters, where APP interacts with β- and γ-secretases to produce the Aβ peptide. When astrocyte cholesterol synthesis is inhibited, cholesterol levels in neurons decrease, leading to the transport of APP out of lipid clusters, where it interacts with α-secretase to generate soluble APP-α, a neuroprotective form of APP. The balance between Aβ and soluble APP-α is regulated by substrate transport. Low cholesterol levels in neurons can inhibit Aβ accumulation and enable astrocytes to regulate Aβ levels through cholesterol signaling [170]. In aging neurons, APP is often abnormally cleaved by enzymes, leading to the accumulation of toxic amyloid beta fragments that disrupt the functioning of neural memory networks. Increased neuronal activity is accompanied by elevated Aβ levels, which subsequently reduce synaptic excitation [171].

In neurodegenerative diseases, disturbances in cellular calcium regulation systems within the cell membrane, endoplasmic reticulum, and mitochondria manifest as synaptic dysfunction, reduced plasticity, and neuronal apoptosis [172]. The β-amyloid peptide, a major component of atherosclerotic plaques in Alzheimer’s disease, may exacerbate neuronal death. Additionally, it can cause oxidative damage to red blood cells by disrupting membrane phospholipids and reducing their volume [172]. Consequently, excessive calcium influx into postsynaptic terminals following NMDA receptor stimulation may indirectly contribute to inflammation and pain hypersensitivity [139]. Notably, the activity of NMDA receptors and calcium ion permeability can be inhibited by extracellular magnesium ions (Mg^2+^). Hyperphosphorylation of the Tau protein results in conformational changes that impair its ability to bind to microtubules. Free monomers of misfolded Tau protein begin to accumulate, forming oligomers and aggregating [168,173]. Oxidized fatty acids, which are products of oxidative stress, promote Tau protein polymerization [174].

Oxidative stress increases Tau protein hyperphosphorylation, disrupting the structure of the neuronal cytoskeleton. After microtubule disassembly, hyperphosphorylated Tau protein accumulates in the form of spiral filaments, blocking intracellular transport and limiting the movement of selected molecules and mitochondria [173,175]. Tauopathies can also disrupt the blood–brain barrier, induced by ongoing chronic inflammation [175].

In Alzheimer’s disease, typical pathologies such as amyloid plaques and neurofibrillary tangles are frequently observed in the hypothalamus [176]. The early clinical stages of Alzheimer’s disease are characterized by hypothalamic atrophy, with a reduction in volume of approximately 10%. Consequently, orexin neurons have been reported to decrease by 40% to 50% in the lateral hypothalamic area [177]. The hypothalamic nuclei, which regulate circadian physiological rhythms, stress responses, and energy homeostasis, are particularly susceptible to proteinopathies and neurodegeneration.

Obesity, insulin resistance, and diabetes significantly and independently increase the risk of Alzheimer’s disease. These metabolic abnormalities serve as premorbid defects that precede the onset of neurological symptoms [178]. While metabolic changes may be linked to hypothalamic damage, not all the signaling pathways involved have been identified. Hypothalamic atrophy may result from exposure to toxins, pathogens, and molecules that penetrate the brain through the fenestrated blood–brain barrier. Additionally, the functional structures of the hypothalamus have a limited number of nerve cells, high levels of activity, and significant energy consumption. The combination of these factors increases the susceptibility of the hypothalamus to age-related changes and neurodegeneration.

Peripheral impairments are likely to promote brain insulin resistance, which can lead to the development of tau pathology and amyloidogenesis [178]. The potential role of brain insulin resistance in the disturbances of glucose homeostasis observed in Alzheimer’s disease patients is supported by the known involvement of insulin and IGF signaling in the regulation of energy metabolism [178]. Insulin acts on the ventromedial hypothalamus to stimulate the sympathetic nervous system, a major regulator of resting metabolic rate. Alzheimer’s disease is characterized by the presence of amyloid β plaques, tau tangles, and inflammation, all of which contribute to cognitive decline.

In Parkinson’s disease, the intraneuronal accumulation and spreading of misfolded α-synuclein (α-syn) is a hallmark characteristic. α-syn oligomers in the extracellular medium significantly inhibit the firing rate of midbrain interneurons and disrupt burst generation and network synchronization. Exogenous α-syn slows down the firing rate of dopaminergic neurons in the substantia nigra, while selectively upregulating Cav2.2 (N-type) channels promoting calcium-dependent dopamine release [179]. This alteration affects the interplay among calcium influx, spontaneous firing, and dopamine release, leading to dopamine accumulation in the extracellular space and intracellular calcium overload [179].

Dysfunctional ionotropic glutamatergic receptors exacerbate calcium signaling, resulting in excitotoxicity [180,181]. Overstimulation of NMDA receptors and calcium overload can ultimately lead to neuronal death and neurodegeneration, which is a feature of Alzheimer’s disease, Parkinson’s disease, Huntington’s disease, and dementia [139]. The NMDA receptor is a crucial ligand-gated ion channel in the central nervous system that responds to the neurotransmitter glutamate, playing a vital role in synaptic plasticity, learning, and memory. When the balance between NMDA receptor activation and Aβ production is disrupted, Aβ deposition can impair synaptic transmission [93]. In response, glial cells and astrocytes become activated, releasing cytokines that further reduce glutamate uptake and impair synaptic transmission. Concurrently, Aβ-induced extracellular glutamate accumulation leads to synaptic pruning, resulting from a reduced density of glutamate receptors and a decreased efficiency in the recovery of this neurotransmitter in the presynaptic area. Tau protein, which is highly expressed in neurons, plays a crucial role in stabilizing microtubules, supporting neuronal development, maintaining neuronal polarity, and facilitating axonal transport [175].

## 10. Therapeutic Perspectives

A growing body of evidence supports the hypothesis that deficiencies in glucose-based energy metabolism contribute significantly to aging and neurodegeneration. As we age, there is an increase in nicotinamide adenine dinucleotide (NAD) deficiency, which leads to compromised cellular respiration, impaired repair processes, and reduced activity of NAD-dependent enzymes. Acetyl-CoA, the end product of glycolysis, fuels cellular respiration, acts as a building block for fatty acids and lipids during lipogenesis, and acts as a donor for protein acetylation [61,130,181,182].Consequently, the decline in oxidative phosphorylation in mitochondria that occurs with age has a detrimental impact on neuronal life and function. Sirtuins utilize the cofactor NAD to catalyze protein deacetylation, which plays a key role in repair and detoxification processes. Age-related NAD deficiency in neuronal cells impedes repair and detoxification processes, exacerbating problems associated with brain aging [61].

This deficiency primarily arises from dysfunction in the kynurenine pathway, which is exacerbated by increasing inflammatory processes in the aging body. In the early subclinical phase of neurodegenerative diseases, the primary therapeutic goal is to restore the minimum physiological levels of energy metabolism and functional activity. This involves regaining control over key cholinergic systems and reducing inflammation, anxiety, and stress. Interventions targeting the fundamental mechanisms of aging may prove beneficial in preventing and treating various age-related pathologies. A crucial aspect of these interventions is restoring glucose metabolism to physiological levels by increasing intracellular NAD levels, typically through the supplementation of its precursors that bypass the kynurenine pathway.

SIRT1, an NAD-dependent deacetylase, has the capacity to modulate and mitigate aging-related pathogenic processes, such as chronic inflammation and neurodegeneration. Under normal physiological conditions, SIRT1 activates a disintegrin and metalloproteinase domain-containing protein 10 (ADAM10), an enzyme responsible for the alpha-secretase cleavage of amyloid precursor protein, thereby reducing amyloid beta production [77]. Thus, restoring NAD levels necessary for these functions could significantly impact brain functionality, providing protection against neurodegenerative processes. The relationships between NAD, NADH, and sirtuin activities suggest that this family of proteins may act as sensors of metabolic and energy status. In conditions of low nutrient availability, normal intracellular NAD levels enable an increase in sirtuin enzymatic activity, leading to the deacetylation of PGC-1α. This process enhances PGC-1α activity, ultimately resulting in an increased number of mitochondria.

Nicotinamide riboside supplementation appears to be the preferred protective therapy because it may help slow down the aging of nerve cells [61,183]. Intracellular NAD and its phosphorylated form, NADP, play a critical role in redox reactions and biosynthesis, including the synthesis of adenosine triphosphate, nucleic acids, steroids, and fatty acids. Additionally, NAD protects against oxidative stress and acts as a co-substrate for numerous enzymes that regulate key cellular processes like gene expression, DNA repair, proteostasis, calcium handling, and cell death. However, we must remember that NAD supplementation alone cannot halt all brain aging processes. Deficiencies in glucose-based energy metabolism linked to brain aging can lead to irreversible neurodegenerative changes and neuronal death. The effects of aging are most pronounced in brain structures with the highest energy demands, particularly in areas related to memory, such as the cortex. Structures like the hippocampus and striatum, which rely heavily on the energy-intensive process of adult neurogenesis, experience significant degradation. This is the reason why NAD supplementation therapy fails in patients with advanced neurodegenerative diseases.

It is important to remember that even in a healthy brain, the uptake of energy substrates and metabolic precursors by neurons is tightly regulated by blood flow, which in turn depends on the current activity of specific brain structures. Therefore, early supplementation with NAD (ideally during the pre-symptomatic period) should be combined with multisensory brain training, physical activity, and the restoration of normal sleep patterns. This multifaceted approach can promote healthy aging in patients.

## Data Availability

Data sharing is not applicable to this article.
